# β-Sitosterol and Gemcitabine Exhibit Synergistic Anti-pancreatic Cancer Activity by Modulating Apoptosis and Inhibiting Epithelial–Mesenchymal Transition by Deactivating Akt/GSK-3β Signaling

**DOI:** 10.3389/fphar.2018.01525

**Published:** 2019-01-08

**Authors:** Zhang-qi Cao, Xue-xi Wang, Li Lu, Jing-wen Xu, Xiao-bin Li, Guang-ru Zhang, Zhan-jun Ma, An-chen Shi, Yan Wang, Yu-jun Song

**Affiliations:** ^1^School of Basic Medical Sciences, Lanzhou University, Lanzhou, China; ^2^Qinghai Hospital of Traditional Chinese Medicine, Xining, China; ^3^The Second Clinical School, Lanzhou University, Lanzhou, China

**Keywords:** β-sitosterol, gemcitabine, pancreatic cancer, apoptosis, EMT, AKT, GSK-3β

## Abstract

β-sitosterol (BS), a major bioactive constituent present in plants, has shown potent anti-cancer activity against many human cancer cells, but its activity in pancreatic cancer (PC) cells has rarely been reported. Gemcitabine (GEM) is one of the first-line drugs for PC therapy, however, the treatment effect is not sustained due to prolonged drug resistance. In this study, we firstly studied the anti-PC activity and the mechanism of BS alone and in combination with GEM *in vitro* and *in vivo*. BS effectively inhibited the growth of PC cell lines by inhibiting proliferation, inducing G0/G1 phase arrest and apoptosis, suppressed the NF- kB activity, and increased expression of the protein Bax but decreased expression of the protein Bcl-2. Moreover, BS inhibited migration and invasion and downregulated epithelial–mesenchymal transition (EMT) markers and AKT/GSK-3β signaling pathways. Furthermore, the combination of BS and GEM exhibited a significant synergistic effect in MIAPaCa-2 and BXPC-3 cells. More importantly, the combined treatment with BS and GEM lead to significant growth inhibition of PC xenografts. Overall, our data revealed a promising treatment option for PC by the combination therapy of BS and GEM.

## Introduction

Pancreatic cancer (PC) is the most lethal cancer characterized by invasive growth and metastasis, and is known as the most malignant tumor with approximately only 5% of survival rates in 5 years (Kamisawa et al., 2016; Elshaer et al., 2017). The low survival rate is primarily because of the insensitivity to most chemotherapies and radiotherapies (Rocha-Lima, 2008). Gemcitabine (GEM) is considered as the first-line chemotherapy drug for patients with PC (Hidalgo, 2010). However, many clinical trials have shown that GEM alone or in combination with other drugs, including cetuximab and erlotinib, exhibits little improvement in overall survival (Antoniou et al., 2014). Therefore, there is an urgent demand to research and develop new drugs or combination therapeutic strategies to treat this fatal disease.

Recently years, a serious of growth studies have been showed that many nature products from plants exhibit an obvious anti-tumor activity, like paclitaxel, docetaxel, teniposide, vinblastin, camptothecin, curcumin and so on. Thus, screening for bioactive antitumor components have become a significant way to develop anti-cancer drugs (Pu et al., 2008). BS is the most abundant phytosterols, similarly with a structure of cholesterol, and widely found in plants and some traditional Chinese medicine (Ye et al., 2015; Chen et al., 2018). Most importantly, it exhibits a significantly anticancer activity (Bin Sayeed and Ameen, 2015).

β-sitosterol restricts proliferation and induces apoptosis in different cancer cell lines, including gastric, colon, prostate, lung, and breast cancer (Shin et al., 2018). Numerous studies have evidenced that the anticancer effect of BS is related to the induction of apoptosis through blockade of multiple cell signaling mechanisms (Bin Sayeed and Ameen, 2015). For instance, anticancer effects of BS are executed via increasing the levels of first apoptotic signal (Fas), caspase-8 activity, β-catenin activity, phosphorylation of extracellular-signal regulating kinase (ERK), p38 mitogen-activated protein kinase (MAPK), and proliferating cell nuclear antigen (PCNA) (Basker et al., 2010). Molecular studies have shown that BS induces endoreduplication in U937 and HL60 cells through the PI3K/Akt and Bcl-2 signaling pathways to promote spindle microtubule dynamics (Moon et al., 2008). Furthermore, BS induces apoptosis in A549 cells by inducing sub-G1 phase arrest (Rajavel et al., 2018). However, the effects of BS in anti-PC activity and the specific mechanisms of these effects remain unclear and have rarely been reported.

Dysregulation of epithelial–mesenchymal transition (EMT) could lead to malignant progression of tumors. EMT separates stationary epithelial cells from each other and converts the cells to a fibroblast-like (mesenchymal) phenotype, with enhanced motility and anti-chemotherapeutic capacity (Marcucci et al., 2016). Altered expression of some specific markers is the characteristic of dysregulated EMT, such as upregulation of Snail, vimentin, and ZEB1 and downregulation of E-cadherin (Savagner, 2010). With respect to the regulatory factors of EMT, Akt and glycogen synthase kinase-3β (GSK-3β) play important roles in regulating EMT (Zhou et al., 2015; Liu et al., 2016). Previous studies have documented that GSK-3β plays a crucial role in regulating cytoskeleton maintenance, migration and invasion, and gene transcription (Liu et al., 2002; Yoshimura et al., 2005; Zhang et al., 2012). With respect to PC, less differentiated phenotypes and poor survival rates are usually associated with dysregulated EMT (Javle et al., 2007). Therefore, targeting EMT is still a promising strategy for the eradication of PC.

In this report, we have investigated the effects of BS alone and in combination with GEM against PC cells, determined the mechanisms of anticancer activity, and tested the inhibition ability of PC growth in BXPC-3 xenograft tumors.

## Materials and Methods

### Chemicals and Reagents

β-sitosterol (98% purity, Yuanye Biotechnology, Shanghai, China) was dissolved in dimethyl sulfoxide (DMSO) to 500 μmol/L and stored for subsequent use (final DMSO concentration < 0.1%). GEM–HCl was obtained from Eli Lilly Company (Indianapolis, IN, United States) and dissolved in normol saline (Sichuan Colen Pharmaceutical Industry, Chengdu, China) to 100 μmol/L and stored. Soybean oil was obtained from Yuanye Biotechnology (Shanghai, China). Rabbit phospho-Akt, anti-Akt, phospho-GSK-3β, anti-GSK-3β, vimentin, Snail, and *E*-cadherin antibodies were bought from Cell Signaling Technology (Danvers, MA, United States). Rabbit phospho-NF-kB p65, anti-NF-kB p65 antibodies were bought from Abcom (Cambridge, United Kingdom). Rabbit anti-Bax, Bcl-2, Ki67, and rabbit anti-GAPDH were purchased from Proteintech (Wuhan, China). BAY11-7082 (BAY) was obtained from Beyotime (Shanghai, China). Perifosine (PER) was purchased from Cell Signaling Technology (Danvers, MA, United States). LiCL was bought from Sigma-Aldrich (St. Louis, MO, United States). Immunohistochemistry (IHC) Detection Kit was obtained from ZSGB-BIO (Beijing, China). Haematoxylin semen was obtained from Beijing Solarbio Science and Technology (Beijing, China). MTT [3-(4,5-dimethylthiazol-2-yl)-2, 5-diphenyltetrazolium bromide] was obtained from Sigma-Aldrich (St. Louis, MO, United States). The 24-well modified Boyden chamber (8-μm pore size) and Matrigel were obtained from Corning Incorporated (New York, NY, United States). The annexin V/propidium iodide (PI) apoptosis kit and Cell cycle detection kit were purchased from Beyotime (Shanghai, China).

### Cell Lines and Cell Culture

Human PC cell lines MIA-PaCa-2 and BXPC-3 were obtained from the Typical Culture Preservation Committee Cell Bank (Shanghai, China). MIA-PaCa-2 and BXPC-3 cells were cultured in RPMI 1640 medium (Hyclone, Carlsbad, CA, United States) containing 10% fetal bovine serum (Hyclone) and 1% penicillin–1% streptomycin (Hyclone). All cell lines were cultured with 5% CO_2_ at 37°C in an incubator.

### Cell Proliferation Assay and Synergy Analyses

MTT assay was performed to detect cell proliferation and viability. MIA-PaCa-2 and BXPC-3 cells (5000 cells/well) were seeded and cultured in 96-well plates with 100 μL of medium, After treatment with different concentrations of BS, GEM, and BS + GEM for various time-points, 20 μL of MTT (5 mg/mL) was added into each well. After incubation at 37°C for 4 h, 100 μL of DMSO was added to each well, and the plates were shaken gently for 6 min. Finally, absorbance values were determined at 490 nm using an ELx800 ELISA reader (Molecular Devices, United States). After plate reading, data were analyzed by CalcuSyn software package version 2.1 (Biosoft, Cambridge, United Kingdom) to calculate the combination index (CI) of different treatments based on the median effect equation. Cell viability was shown as percent cell viability correlated with the vehicle-treated group.

### Fluorescence Microscopic Analysis of Apoptosis

MIA-PaCa-2 and BXPC-3 cells were seeded in 6-well culture plates and cultured for 24 h. After treatment with various concentrations of BS, GEM, and BS + GEM for 48 h, cells were incubated in 4% paraformaldehyde for 10 min at room temperature (24–26°C), after which the buffer was decanted and cells were washed three times with cold PBS, and incubated with Hoechst 33258 (5 mg/mL) for 25 min in the dark at room temperature. After washing with cold PBS, we observed the morphological changes in the cells, including smaller dense bodies emitting bright blue fluorescence and a stained nucleus with condensed chromatin, and photographed at 200X magnification under a fluorescence microscope (Olympus, Yokohama, Japan).

### Cell Apoptosis Assay by Flow Cytometry

MIA-PaCa-2 and BXPC-3 cells were seeded in 25-cm^2^ culture dishes, and when the cell density reached 70% confluency, the culture medium was replaced with fresh medium containing different concentrations of BS, GEM, and BS + GEM and incubated for 48 h. The cells obtained were resuspended in 195 μL of 1× binding buffer containing PI (10 μL) and annexin V–fluorescein isothiocyanate (FITC) (5 μL), and cells were incubated for 15 min in the dark. The results were immediately analyzed by flow cytometry (BD Bioscience, Bedford, MA, United States).

### Cell Cycle Analysis

To further confirm apoptosis, cellular DNA was subjected to flow cytometry analysis. MIA-PaCa-2 and BXPC-3 cells were seeded in 25-cm^2^ culture dishes. When the cell density reached 70% confluency, the culture medium was replaced with fresh medium containing different concentrations of BS, GEM, and BS + GEM for 48 h. After treatment, the cells obtained were washed two times with cold PBS and fixed in 70% ice-cold ethanol overnight at 4°C. The fixed cells were centrifuged for 3 min at 1200 rpm, the supernatant was decanted, and the pellet was washed two times with PBS. Before analysis, the cells were washed again with PBS and stained with 50 mg/mL PI and incubated in the dark at room temperature for 30 min. Finally, the cell cycle was evaluated immediately through flow cytometry (BD Bioscience, Bedford, MA, United States).

### Cell Migration Assay

Migration assay was performed using 8-μm pore size 24-well transwell chambers (Corning Inc., New York, NY, United States). In brief, 8 × 10^4^ MIA-PaCa-2 and BXPC-3 cells resuspended in 100 μL of non-serum culture medium containing different concentrations of BS, GEM, and BS + GEM were placed in the upper chamber of the insert and 650 μL of the medium with 10% FBS was used as the chemoattractant in the lower chamber. Cells were allowed to migrate for 48 h and then detached from the top surface of the membrane with a cotton swab. The cells on the bottom surface of the membrane were fixed with 4% paraformaldehyde for 20 min and then stained with 0.2% crystal violet for 20 min.

### Cell Invasion Assay

The upper chamber of the transwell plate was coated with Matrigel (Corning Inc, New York, NY, United States) and incubated overnight. MIA-PaCa-2 and BXPC-3 cells (8 × 10^4^) were starved for 12 h and placed into the upper chamber with varying concentrations of BS, GEM, and BS + GEM for 48 h. The lower chambers were supplemented with 650 μL of the medium supplemented with 10% FBS. After incubation for 48 h, the upper chambers were removed, and the cells migrated into the lower chambers were fixed with 4% paraformaldehyde for 20 min and then stained with 0.2% crystal violet for 20 min.

### Protein Extraction and Western Blot

Total proteins were extracted, lysed in RIPA buffer, and quantified with the BCA protein assay kit (Beyotime, Shanghai, China) according to the manufacturer’s instruction. In brief, proteins were separated on sodium dodecyl sulfate-polyacrylamide gel electrophoresis (SDS-PAGE; 10–15%) gel, electrically transferred onto polyvinylidene fluoride (PVDF) membranes and blocked for 2 h in blocking buffer [0.1% Tween 20 in Tris-buffered saline (TBST) and 5% bovine serum albumin] at room temperature. After washing three times with TBST, the primary antibodies were separately incubated with the membranes overnight at 4°C. Next, the corresponding secondary antibodies were incubated with the membranes for 1 h. Finally, the membranes were visualized using chemiluminescence (Bio-Rad, United States). Western blot data were analyzed by the ImageJ software.

### *In vivo* Analysis of the Combination Drug Effect

All experiments were approved by the Lanzhou University Animal Ethics Committee and were performed in accordance with the National Institutes of Health Guidelines for Animal Care. Female BALB/c mice (nu/nu; 5-weeks-old; 19–23 g weight) were obtained from the Shanghai SLAC Animal Center (Shanghai, China). These nude mice were bred in specific pathogen-free (SPF) conditions, with stable humidity and temperature (24–26°C) under a 12-h light/dark cycle. BXPC-3 cells (0.2 mL; 7 × 10^6^ cells) were subcutaneously injected into the right flank of the nude mice. After the tumor volume reached approximately 90 mm^3^, the mice were randomly divided into four groups according to treatment: (1) control group (vehicle; soybean oil, once a day, intraperitoneally); (2) BS group (80 mg/kg, once a day, intraperitoneally); (3) GEM group (100 mg/kg, once every 3 days, intraperitoneally); and (4) combination group (80 mg/kg BS, once a day and 100 mg/kg GEM, once every 3 days, intraperitoneally). Tumor weight and dimensions (length and width) were measured individually using an electronic scale and a Vernier caliper every 2 days. The tumor volume (mm^3^) was calculated as V = (length/2) × (width^2^). After 28 days, the mice were sacrificed, and the tumors were removed, weighed, and prepared for paraffin embedment.

### TUNEL Assay

Apoptotic cells in BXPC-3 tumor xenograft tissue were detected by TUNEL (terminal deoxynucleotidyltransferase-mediated dUTP nick end-labeling) using a commercially available kit (Promega, Beijing, China). In brief, 3-μm thick sections obtained from the paraffin-embedded tissue were dewaxed two times using xylene for 15 min, hydrated using an ethanol gradient (twice with 100% for 5 min, then 85% for 5 min, and 75% for 5 min), fixed in 4% formaldehyde solution at room temperature for 20 min, and incubated with proteinase K at 37°C for 30 min. The TUNEL assay kit containing TdT was prepared immediately before use according to the manufacturer’s protocol. After washing with PBS, the sections were counterstained with DAPI (4′,6-diamidino-2-phenylindole). Apoptotic cells in the sections were observed and photographed at 200X magnification under a fluorescence microscope (Olympus, Yokohama, Japan).

### Haematoxylin–Eosin (HE) Staining

Tumor xenograft tissues were embedded in paraffin and sliced into 4-μm sections for HE staining. The sections were dyed with haematoxylin semen for 3 min, washed with tap water for 15 s, and stained with 1% hydrochloric acid ethanol for 15 s. After washing with distilled water for 1 min, the sections were dyed with eosin for 50 s, followed by light washing with distilled water for 15 s. The sections were dehydrated with gradient ethanol and soaked in xylene and sealed with neutral balsam. Images were photographed using an optical microscope at 200X magnification (Olympus, Yokohama, Japan).

### Immunohistochemical Analysis

Tumor xenograft tissues were embedded in paraffin, sliced into 4-μm sections in for IHC staining, dewaxed, rehydrated, immersed in citrate buffer for antigen retrieval at 95°C for 10 min, and then peroxidase inhibitor was added for 10 min. Next, the sections were incubated with primary antibodies at 4°C overnight. A suitable secondary antibody was incubated with the tissue sections for 40 min at room temperature and washed with PBS and incubated with diaminobenzidine (DAB) for 10 min, followed by subsequent haematoxylin staining. Images were photographed using an optical microscope at 200X magnification (Olympus, Yokohama, Japan).

### Statistical Analysis

Data are represented as mean ± standard deviation of three independent experiments. The control and test groups were analyzed by the pair-wise two-sample *t*-test. SPSS 21.0 (IBM, United States) was used to analyze statistical data. All data are depicted as mean ± SD. ^∗^Indicates the combination, BS, or GEM group compared to the control group alone; ^+^indicates the BS group compared to the combination group; ^#^indicates GEM group compared to the combination group. ^∗^*P* < 0.05, ^∗∗^*P* < 0.01, ^∗∗∗^*P* < 0.001, ^+^*P* < 0.05, ^++^*P* < 0.01, ^+++^*P* < 0.001; ^#^*P* < 0.05, ^##^*P* < 0.01, ^###^*P* < 0.001.

## Results

### BS Effectively Inhibits Proliferation of PC Cells

**FIGURE 1:**
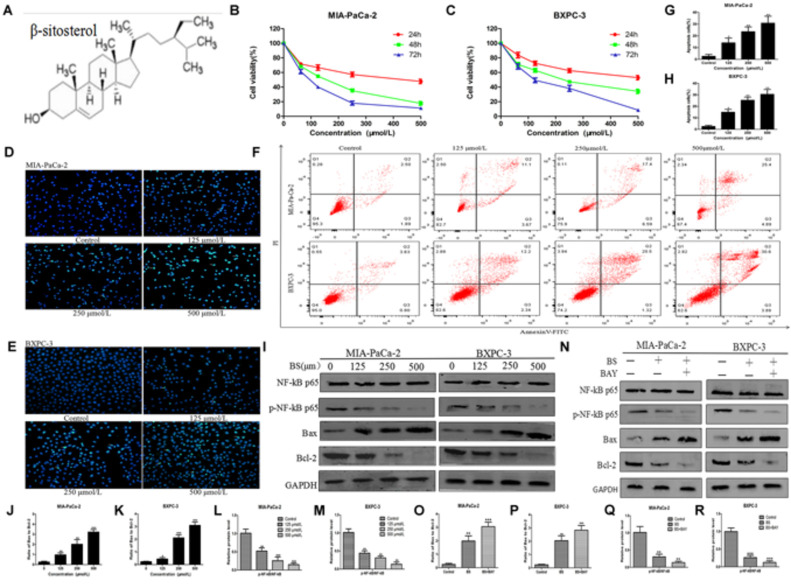
β-sitosterol (BS) effectively inhibits proliferation and induces apoptosis of pancreatic cancer cells. **(A)** Chemical structure of the indicated drug. **(B,C)** MIA-PaCa-2 and BXPC-3 cells were treated with various concentration of BS for 24, 48, and 72 h, and the negative control group was treated with an equal volume of medium containing DMSO. Cell viabilities were detected with the MTT assay. All data are depicted as mean ± SD (*n* = 5). **(D,E)** MIA-PaCa-2 and BXPC-3 cells were treated with different concentrations of BS for 48 h. Morphological changes in MIA-PaCa-2 and BXPC-3 cells were observed at magnification of 200X. The arrows indicate several apoptotic cells that were shrunken, with fragmented or condensed nucleus and improved brightness. **(F–H)** MIA-PaCa-2 and BXPC-3 cells were treated with different concentrations of BS for 48 h. Flow cytometry analysis of BS-induced apoptosis of MIA-PaCa-2 and BXPC-3 cells by annexin V–fluorescein isothiocyanate (FITC)/propidium iodide (PI) staining. All data are depicted as mean ± SD (*n* = 3; ^∗^*P* < 0.05; ^∗∗^*P* < 0.01). **(I–M)** MIA-PaCa-2 and BXPC-3 cells were treated with various concentrations of BS for 48 h. The expressions of NF-kB p-65, p-NF-kB p-65, Bax and Bcl-2 in Miapaca-2 and Bxpc-3 cells were analyzed by western blotting, and GAPDH was used as the control. The relative protein levels of the ratio p-NF-kB p-65 to NF-kB p-65, Bax to Bcl-2 were shown in the histograms. All data are depicted as mean ± SD (*n* = 3; ^∗^*P* < 0.05; ^∗∗^*P* < 0.01; ^∗∗∗^*P* < 0.001). **(N–R)** MIA-PaCa-2 and BXPC-3 cells were treated with just culture medium, BS (250 μM/L), or both BS (250 μM/L) and BAY (10 μM/L). The expressions of Bax, Bcl-2, NF-kB p-65 and p-NF-kB p-65 in MIA-PaCa-2 and BXPC-3 cells were tested by western blotting, the relative protein levels of the ratio p-NF-kB p-65 to NF-kB p-65, Bax to Bcl-2 were shown in the histograms. All data are depicted as mean ± SD (*n* = 3; ^∗^*P* < 0.05; ^∗∗^*P* < 0.01; ^∗∗∗^*P* < 0.001).

The chemical structure of BS is shown in Figure 1A. To determine the effect of BS in PC cells, MIA-PaCa-2 and BXPC-3 were treated with various concentrations of BS (0, 62.5, 125, 250, and 500 μM/L) for 24, 48, and 72 h. Cell viabilities were determined by the MTT assay for each indicated dose and time point. As expected, treatment with BS resulted in reduced viability of Miapaca-2 and Bxpc-3 cells in a concentration-dependent and time-dependent manner (Figures 1B,C). The IC_50_ values after treatment with BS for 24, 48, and 72 h were 248.6 ± 3.96 μM, 210.1 ± 1.33 μM, and 127.6 ± 0.61 μM, respectively, in Miapaca-2 cells, whereas the values were 434.2 ± 4.17 μM, 218.3 ± 1.37 μM, and 126.2 ± 0.71 μM, respectively, in BXPC-3 cells.

### BS Induces Apoptosis in PC Cells

To investigate whether the anti-proliferative activity of BS is related to apoptosis, Hoechst 33258 staining assay was performed. After treatment with different concentrations of BS for 48 h, cell morphology was observed using a fluorescence microscope. In the control group, the cell nucleus was completely stained and round, indicating viable cells. However, when treated with BS, apoptosis characteristics, including shrunken cell, fragmented or condensed nucleus, and improved brightness, were observed in MIA-PaCa-2 and BXPC-3 cells (Figures 1D,E). To further verify the apoptotic effect of BS, cells were labeled with PI and annexin V. Flow cytometry analysis revealed that apoptotic cell numbers in BS-treated groups increased evidently in a dose-dependent manner compared to those in untreated groups (Figures 1F–H). Moreover, we examined the levels of the pro-apoptotic protein Bax, anti-apoptotic protein Bcl-2, protein NF-kB p-65 and protein p-NF-kB p-65 by western blot. The results showed that BS upregulated Bax levels but downregulated Bcl-2 and p-NF-kB p-65 levels, whereas it exhibited no effect on the total level of NF-kB p-65 (Figures 1I,L,M). Comprehensively, the ratio of Bax to Bcl-2 increased in the BS-treatment group (Figures 1J,K). To confirm whether BS mediated the cell behavior involve with the NF-kB signaling way, MIA-PaCa-2 and BXPC-3 were treated with just culture medium, BS (250 μM/L), or both BS (250 μM/L) and BAY (10 μM/L). Western blot was performed to assess whether NF-kB inhibitor could affect the ratio of Bax to Bcl-2. The results showed that the BAY increase the ratio of Bax to Bcl-2, decrease the expression of protein p-NF-kB p-65, whereas it exhibited no effect on the total level of NF-kB p-65 (Figures 1O–R). These results demonstrate that BS could induce apoptosis by NF-kB pathway in PC cells.

### BS Affects Cell Cycle Progression in PC Cells

**FIGURE 2:**
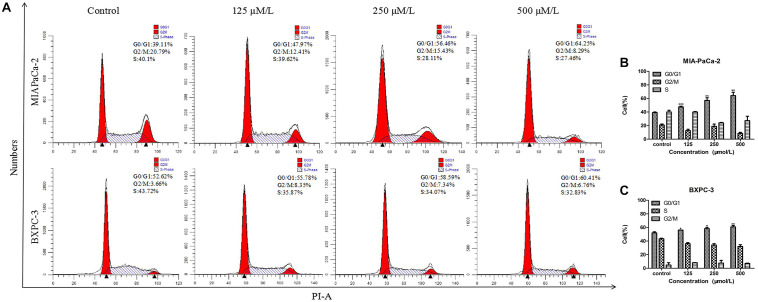
β-Sitosterol (BS) affects cell cycle progression in pancreatic cancer cells. **(A–C)** MIA-PaCa-2 and BXPC-3 cells were treated with different concentrations of BS for 48 h. G0/G1 cell cycle arrest were observed in MIA-PaCa-2 and BXPC-3 cells. All data are depicted as mean ± SD (*n* = 3; ^∗^*p* < 0.05; ^∗∗^*p* < 0.01; ^∗∗∗^*p* < 0.001).

The DNA duplication process in cells is regarded as the cell cycle. Cell cycle arrest inhibits cancer cell growth and may provide a significant strategy for the treatment of cancer (Bartek and Lukas, 2001a,b). To detect whether the cytotoxicity of BS was attributed to changes in the cell cycle, MIA-PaCa-2 and BXPC-3 cells were treated with different concentrations of BS for 48 h and analyzed by flow cytometry. It was observed that cell cycle distribution in the G0/G1 phase was augmented as BS concentration increased (Figures 2A–C), indicating that BS could induce G0/G1 phase arrest in PC cells.

### BS Decreases Migration and Invasion by PC Cells

**FIGURE 3:**
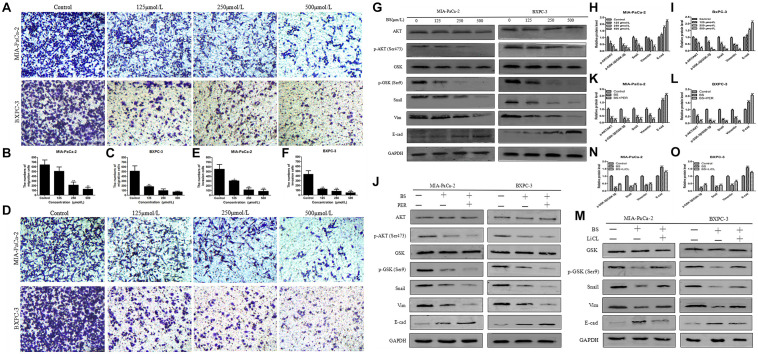
β-Sitosterol (BS) decreases migration and invasion and downregulates the expression of epithelial–mesenchymal transition (EMT) markers and AKT/GSK-3β signaling pathways in pancreatic cancer cells. **(A–C)** For transwell migration assays, MIA-PaCa-2 and BXPC-3 cells were treated with various concentrations of BS for 48 h. The number of cells were counted under a microscope (200× magnification). Quantification results are showed for migration of MIA-PaCa-2 and BXPC-3 cells. All data are depicted as mean ± SD (*n* = 3; ^∗^*p* < 0.05; ^∗∗^*p* < 0.01). **(D–F)** For Matrigel-coated invasion assays, MIA-PaCa-2 and BXPC-3 cells were treated with various concentrations of BS for 48 h. The number of cells were counted under a microscope (200× magnification). Quantification results are shown for invasion by MIA-PaCa-2 and BXPC-3 cells. All data are depicted as mean ± SD (*n* = 3; ^∗^*p* < 0.05; ^∗∗^*p* < 0.01). **(G–I)** MIA-PaCa-2 and BXPC-3 cells were treated with various concentrations of BS for 48 h, and the expression levels of Akt, p-Akt, GSK-3β, p-GSK-3β, Snail, vimentin, and E-cadherin were detected by western blotting. The relative protein levels of p-Akt/Akt, p-GSK-3β/GSK-3β, Snail, vimentin, and E-cadherin in MIA-PaCa-2 and BXPC-3 cells were shown in the histograms. All data are depicted as mean ± SD (*n* = 3; ^∗^*p* < 0.05; ^∗∗^*p* < 0.01; ^∗∗∗^*p* < 0.001). **(J–L)** MIA-PaCa-2 and BXPC-3 cells were treated with just culture medium, BS (250 μM/L), or both BS (250 μM/L) and PER (10 μM/L). The expressions of Akt, p-Akt, GSK-3β, p-GSK-3β, Snail, vimentin, and E-cadherin in MIA-PaCa-2 and BXPC-3 cells were tested by western blotting, the relative protein levels of p-Akt/Akt, p-GSK-3β/GSK-3β, Snail, vimentin, and E-cadherin were shown in the histograms. All data are depicted as mean ± SD (*n* = 3; ^∗∗^*p* < 0.01; ^∗∗∗^*p* < 0.001). **(M–O)** MIA-PaCa-2 and BXPC-3 cells were treated with just culture medium, BS (250 μM/L), or both BS (250 μM/L) and LiCL (20 mM/L). The expressions of GSK-3β, p-GSK-3β, Snail, vimentin, and E-cadherin in MIA-PaCa-2 and BXPC-3 cells were tested by western blotting, the relative protein levels of p-GSK-3β/GSK-3β, Snail, vimentin, and E-cadherin were shown in the histograms. All data are depicted as mean ± SD (*n* = 3; ^∗∗^*p* < 0.01; ^∗∗∗^*p* < 0.001).

Cell migration is induced or initiated by triggering receptors that stimulate subcellular organelle reordering and cytoskeletal remodeling. EMT is increasingly regarded as a mechanism by which cells obtain the necessary features of migration and invasion in malignant tumors (Huber et al., 2005; Guarino, 2007). To test whether BS could inhibit migration and invasion by PC cells, transwell assays were conducted. We noticed that BS significantly inhibited migration of both MIA-PaCa-2 and BXPC-3 cells in a dose-dependent manner (Figures 3A–C). Consistently, invasion by MIA-PaCa-2 and BXPC-3 cells diminished substantially with increasing concentrations of BS (Figures 3D–F). These findings suggested that BS could further suppress migration and invasion by PC cells.

### BS Downregulates the Expression of EMT Markers and AKT/GSK-3β Signaling Pathway in PC Cells

Because the malignancy of PC is strongly related with EMT (Thomas et al., 2018) and we found that BS functionally suppressed migration and invasion by PC cells, we then investigated whether BS could inhibit EMT. MIA-PaCa-2 and BXPC-3 cells were treated with different concentrations of BS (0, 125, 250, 500 μM/L) for 48 h. Western blot was performed to assess whether BS could affect the expression of specific EMT markers. Consistent with the results of invasion and migration experiments, the result demonstrated that BS dose-dependently reduced the expression of Snail and vimentin, whereas it increased the expression of E-cadherin (Figures 3G–I). The Akt/GSK-3β signaling pathway plays an important role in the regulation of EMT in tumor progression (Liu et al., 2014). To explore the effect of BS on Akt and GSK-3β activation, we treated MIA-PaCa-2 and BXPC-3 cells with various concentrations of BS (0, 125, 250, 500 μM/L) for 48 h. Western blotting revealed that BS dose-dependently reduced phospho-Akt and phospho-GSK-3β levels, whereas it exhibited no effect on the total level of AKT and GSK-3β (Figures 3G–I). To further examine the role of AKT/GSK-3β pathway in BS-mediated inhibition of EMT in PC cells, PER, an inhibitor of AKT, was used to deactivate the activation of AKT. MIA-PaCa-2 and BXPC-3 cells were treated with just culture medium, BS (250 μM/L), or both BS (250 μM/L) and PER (10 μM/L), western blot revealed that the PER reduced the protein level of phospho-Akt, phospho-GSK-3β, Snail and vimentin, whereas it increased the protein level of E-cadherin, but it exhibited no effect on the total level of AKT and GSK-3β (Figures 3J–L). Additionally, LiCL, an GSK-3β inhibitor, was used to investigate the inhibition ability of BS involved in EMT, MIA-PaCa-2 and BXPC-3 cells were treated with just culture medium, BS (250 μM/L), or both BS (250 μM/L) and LiCL (20 mM/L), western blot demonstrated that LiCL increased the expression of phospho-GSK-3β, Snail and vimentin, whereas it decreased the expression of E-cadherin, but it exhibited no effect on the total level of GSK-3β (Figures 3M–O). Taken together, these results suggested that the Akt/GSK-3β pathway participate in BS-inhibited EMT in PC cells.

### Combination of BS and GEM Synergistically Inhibited Proliferation of PC Cells

**FIGURE 4:**
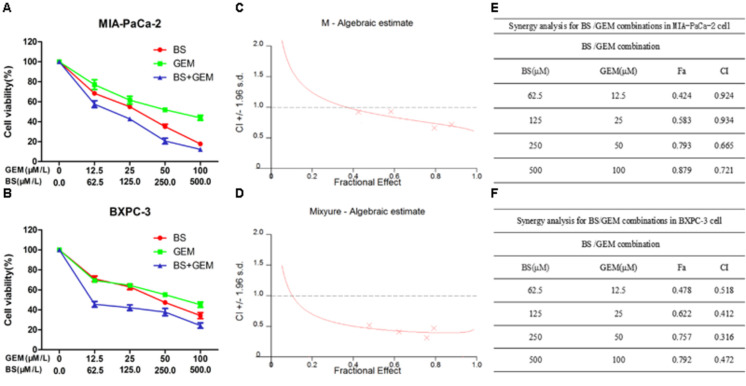
Combination of β-sitosterol (BS) and gemcitabine (GEM) synergistically inhibited proliferation of pancreatic cancer cells. **(A,B)** MIA-PaCa-2 and BXPC-3 cells were treated with different concentrations of BS (0, 62.5, 125, 250, and 500 μM/L), GEM (0, 12.5, 25, 50, and 100 μM/L), or both for 48 h. Cell viabilities were then detected by the MTT assay. **(C,D)** BS/GEM combination algebraic estimate calculated in MIA-PaCa-2 and BXPC-3 cells by the CalcuSyn software. **(E,F)** Tables show the fraction affected (Fa) and combination indexes (CIs) of BS/GEM at the indicated dose levels in MIA-PaCa-2 and BXPC-3 cells.

To investigate whether BS and GEM exhibited synergistic inhibition of cell proliferation, we detected the anticancer activity of the combination group by a cell viability assay in PC cells. Cells were treated with various concentrations of BS (0, 62.5, 125, 250, 500 μM/L), GEM (0, 12.5, 25, 50, 100 μM/L), or both for 48 h. Compared with either agent alone treatment, the combination treatment exhibited higher inhibition of cell growth (Figures 4A,B). To identify the synergistic effects of BS and GEM, CIs were calculated by the CalcuSyn software for each dose combination. Values of CI > 1 indicated antagonism, =1 indicated additive effect, and <1 indicated synergy. Different degrees of synergistic effects were observed in PC cells. As shown in Figures 4C,D, treatment of MIA-PaCa-2 and BXPC-3 cells with BS in combination with GEM resulted in completely low CI values of <1, which is a characteristic of synergistic effect (Figures 4E,F). Notably, BS in combination with GEM produced significant synergistic effects, with CI values < 1 for the combination of 250 μM/L BS and 50 μM/L GEM in MIA-PaCa-2 (CI = 0.665) and BXPC-3 (CI = 0.316) cells.

### Combination of BS and GEM Synergistically Induces Apoptosis of PC Cells

**FIGURE 5:**
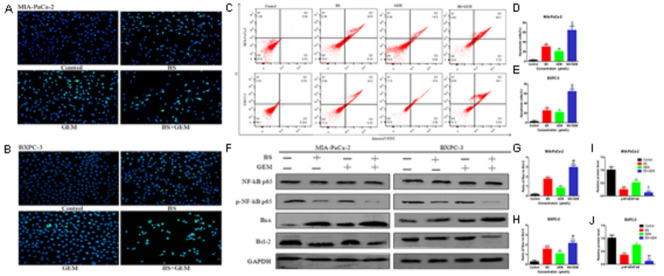
Combination of β-sitosterol (BS) and gemcitabine (GEM) synergistically induced apoptosis in pancreatic cancer cells. MIA-PaCa-2 and BXPC-3 cells were treated with BS (250 μM/L) and GEM (50 μM/L) alone and in combination for 48 h. **(A,B)** Morphological changes in MIA-PaCa-2 and BXPC-3 cells were observed at magnification of 200X. The arrows indicate several apoptotic shrunken cells, with fragmented or condensed nucleus and improved brightness. **(C–E)** Flow cytometry analysis revealed that BS and GEM alone and in combination induced apoptosis of MIA-PaCa-2 and BXPC-3 cells, as determined by annexin V–fluorescein isothiocyanate (FITC)/propidium iodide (PI) staining. All data are depicted as mean ± SD (*n* = 3; ^∗^*P* < 0.05; ^∗∗^*P* < 0.01; ^+^*P* < 0.01; ^++^*P* < 0.01; ^##^*P* < 0.01). **(F–J)** The expressions of NF-kB p-65, p-NF-kB p-65, Bax and Bcl-2 in Miapaca-2 and BxPC-3 cells were determined by western blotting. The relative protein levels of the ratio p-NF-kB p-65 to NF-kB p-65, Bax to Bcl-2 were shown in the histograms. All data are depicted as mean ± SD (*n* = 3; ^∗^*P* < 0.01; ^∗∗^*P* < 0.001; ^∗∗∗^*P* < 0.001; ^+^*P* < 0.01; ^++^*P* < 0.001; ^##^*P* < 0.01; ^###^*P* < 0.001).

To examine whether the combination of BS and GEM induced apoptosis in PC cells, the Hoechst 33258 staining assay was performed. After MIA-PaCa-2 and BXPC-3 cells were treated with BS (250 μM/L) and GEM (50 μM/L) alone or in combination for 48 h, cell morphology was observed using a fluorescence microscope. The result showed that the combination treatment lead to higher characteristics of apoptosis than that by either of the agents alone (Figures 5A,B). To further verify the apoptotic effect, the cells were labeled with PI and annexin V Flow cytometry analysis revealed that compared to BS or GEM alone, the number of apoptotic cells increased significantly in the combination group (Figures 5C–E). Moreover, we examined the levels of the pro-apoptotic protein Bax, anti-apoptotic protein Bcl-2, protein NF-kB p-65 and protein p-NF-kB p-65 by western blot. The results showed that the combination treatment significantly upregulated Bax levels but downregulated Bcl-2 and p-NF-kB p-65 levels, whereas it exhibited no effect on the total level of NF-kB p-65 (Figures 5F–J). These results demonstrate that the combination treatment group induced apoptosis more obviously in PC cells compared to that by the groups treated with only one of the agents.

### Combination of BS and GEM Affects Cell Cycle Progression in PC Cells

**FIGURE 6:**
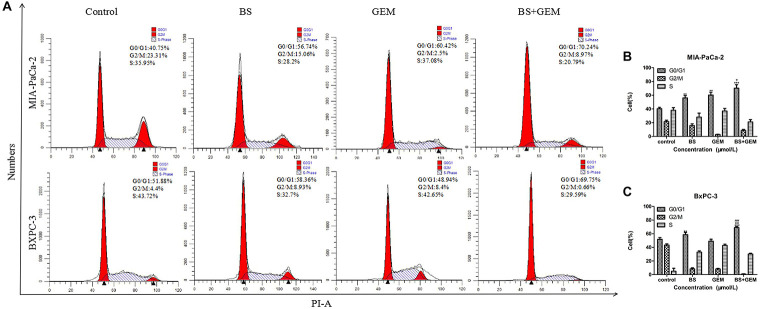
Combination of β-sitosterol (BS) and gemcitabine (GEM) affect cell cycle progression of pancreatic cancer cells. MIA-PaCa-2 and BXPC-3 cells were treated with BS (250 μM/L) and GEM (50 μM/L) alone and in combination for 48 h and analyzed by flow cytometry. **(A–C)** Cell cycle distribution in the G0/G1 phase was observed to be augmented in the combination group compared with either one of the agents group. All data are depicted as mean ± SD (*n* = 3; ^∗∗^*p* < 0.01; ^∗∗∗^*p* < 0.001; ^+^*p* < 0.05; ^ + +^ *p* < 0.001; ^###^*p* < 0.001).

To determine whether the cytotoxicity of combination of BS with GEM was attributed to its ability to affect the cell cycle, MIA-PaCa-2 and BXPC-3 cells were treated with BS (250 μM/L) and GEM (50 μM/L) alone and in combination for 48 h and analyzed by flow cytometry. Cell cycle distribution in the G0/G1 phase was observed to be augmented in the combination group compared with only one of the agents group (Figures 6A–C), indicating that combination of BS and GEM could significantly induce G0/G1 phase arrest in PC cells.

### Combination of BS and GEM Synergistically Decreases Migration and Invasion by PC Cells

**FIGURE 7:**
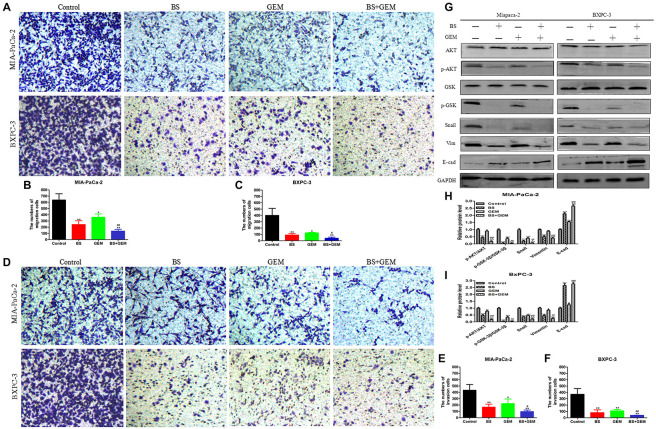
Combination of β-sitosterol (BS) and gemcitabine (GEM) synergistically decrease migration and invasion and downregulate the expression of epithelial–mesenchymal transition (EMT) markers and AKT/GSK-3β signaling pathways in pancreatic cancer cells. **(A–C)** For transwell migration assays, MIA-PaCa-2 and BXPC-3 cells were treated with BS (250 μM/L) and GEM (50 μM/L) alone and in combination for 48 h. The number of cells were counted under a microscope (200× magnification). Quantification results are shown for migration of MIA-PaCa-2 and BXPC-3 cells. All data are depicted as mean ± SD (*n* = 3; ^∗^*p* < 0.05; ^∗∗^*p* < 0.01; ^#^*p* < 0.05; ^##^*p* < 0.01). **(D–F)** For Matrigel-coated invasion assays, MIA-PaCa-2 and BXPC-3 cells were treated with BS (250 μM/L) and GEM (50 μM/L) alone and in combination for 48 h. The number of cells was counted under a microscope (200× magnification). Quantification results are shown for invasion by MIA-PaCa-2 and BXPC-3 cells. All data are depicted as mean ± SD (*n* = 3; ^∗^*p* < 0.05; ^∗∗^*p* < 0.01; ^#^*p* < 0.05; ^##^*p* < 0.01). **(G–I)** MIA-PaCa-2 and BXPC-3 cells were incubated with BS (250 μM/L) and GEM (50 μM/L) alone and in combination for 48 h. The expression levels of Akt, p-Akt, GSK-3β, p-GSK-3β, Snail, vimentin, and E-cadherin were detected by western blotting. the relative protein levels of p-Akt/Akt, p-GSK-3β/GSK-3β, Snail, vimentin, and E-cadherin were shown in the histograms. All data are depicted as mean ± SD (*n* = 3; ^∗^*p* < 0.05; ^∗∗^*p* < 0.01; ^∗∗∗^*p* < 0.001; ^+^*p* < 0.05; ^ + +^ *p* < 0.001; ^##^*p* < 0.01; ^###^*p* < 0.001).

To test whether combination of BS and GEM could inhibit migration and invasion by PC cells, transwell assays were conducted. MIA-PaCa-2 and BXPC-3 cells were incubated with BS (250 μM/L) and GEM (50 μM/L) alone and in combination for 48 h. We observed that the combination of BS and GEM strongly inhibited migration of both MIA-PaCa-2 and BXPC-3 cells compared with that by the groups that were treated with either one of the agents (Figures 7A–C). Consistently, invasion by MIA-PaCa-2 and BXPC-3 cells diminished substantially with the combination treatment (Figures 7D–F). These findings suggested that the combination treatment can further inhibit migration and invasion by PC cells.

### Combination of BS and GEM Downregulates the Expression of EMT Markers and AKT/GSK3β Signaling Pathway in PC Cells

To examine whether BS potentiated the antitumor effect of GEM associated with the expression of specific EMT markers, we treated MIA-PaCa-2 and BXPC-3 cells with BS (250 μM/L) and GEM (50 μM/L) alone and in combination for 48 h. Consistent with the results of invasion and migration experiments, western blot demonstrated that the combination treatment significantly reduced the expression of vimentin and Snail, whereas it increased the expression of *E*-cadherin (Figures 7G–I). Western blot also revealed that the combination treatment significantly reduced phospho-Akt and phospho-GSK-3β levels, whereas it exhibited no effect on the total level of AKT and GSK-3β in any of the treatment groups (Figures 7G–I). Taken together, these results suggested that the combination treatment significantly downregulated the Akt/GSK-3β pathway and EMT markers in PC cells.

### Combination of BS and GEM Suppresses Tumor Growth in Xenograft Tumor Models

**FIGURE 8:**
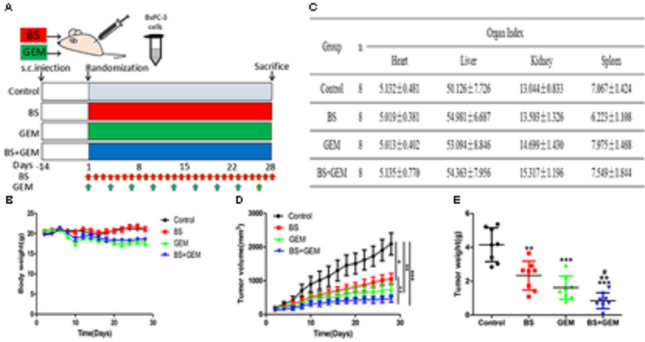
Combination of β-sitosterol (BS) and gemcitabine (GEM) suppressed tumor growth in xenograft tumor models. **(A)** The timeline for generation of xenograft model in nude mice and the treatment groups. Red arrows, BS injections; green arrows, GEM injections; blue arrows, the combination injections of β-sitosterol and gemcitabine. **(B)** Mice body weight was measured on the indicated days. **(C)** Effect of BS and GEM alone and in combination on organ index. All data are depicted as mean ± SD (*n* = 8). **(D)** Tumor volumes were measured on the indicated days. All data are depicted as mean ± SD (*n* = 3; ^∗^*P* < 0.05; ^∗∗^*P* < 0.01; ^∗∗∗^*P* < 0.001; ^++^*P* < 0.01). **(E)** Tumour weight was measured on the indicated days. All data are depicted as mean ± SD (*n* = 3; ^∗∗^P < 0.01; ^∗∗∗^P < 0.001; ^++^*P* < 0.01; ^#^*P* < 0.05).

To further confirm the anti-tumor efficacy of the combination of BS and GEM, we evaluated the ability of the combination treatments group in a xenograft tumor model. We generated xenograft tumors by injecting BXPC-3 cells into BALB/c nude mice and then treated the mice by intraperitoneal injections of BS and GEM alone and in combination, which was initiated at 14 day after tumor cell implantation and was continued up to 28 days (Figure 8A). The body weight and tumor diameters were measured at 2-day intervals. The result showed that body weight did not differ significantly among the combination treatment group and other groups, indicating that the treatments were well tolerated and had no harmful effect on the animals (Figure 8B). The organ indexes for the heart, liver, kidney, and spleen were generally similar between all treatment and control groups (Figure 8C). Moreover, we found that the tumor volume increased rapidly in the control group, but delayed growth in the groups treated with BS or GEM alone, especially delayed growth in combination group (Figure 8D). The tumor weight was reduced by 48.92% and 63.85% in the BS-treated and GEM-treated groups, respectively. Importantly, the combination treatment group further suppressed tumor growth compared to that other treatment groups, with the tumor weight being reduced by 77.25% (Figure 8E).

**FIGURE 9:**
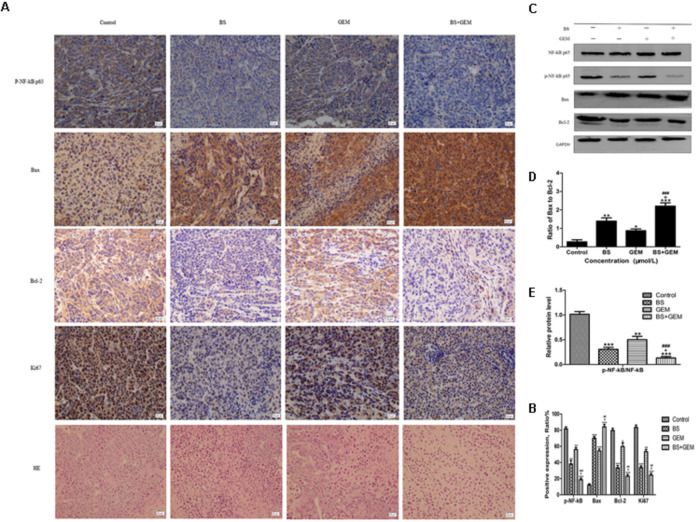
Combination of β-sitosterol (BS) and gemcitabine (GEM) suppressed tumour growth in xenograft tumor model. **(A,B)** Tumor tissues were immunohistochemically stained using p-NF-kB p-65, Bax, Bcl-2, Ki67 and HE, the relative levels of p-NF-kB p-65, Bax, Bcl-2, ki67 were shown in the histograms. All data are depicted as mean ± SD (*n* = 3; ^∗^*P* < 0.01; ^∗∗^*P* < 0.001; ^∗∗∗^*P* < 0.001; ^+^*P* < 0.01; ^##^*P* < 0.001; ^###^*P* < 0.001). **(C–E)** Mice tumors was assessed by western blotting for determining NF-kB p-65, p-NF-kB p-65, Bax and Bcl-2 levels. The relative protein levels of the ratio p-NF-kB p-65 to NF-kB p-65, Bax to Bcl-2 were shown in the histograms. All data are depicted as mean ± SD (*n* = 3; ^∗∗^*P* < 0.001; ^∗∗∗^*P* < 0.001; ^+^*P* < 0.01; ^###^*P* < 0.001).

**FIGURE 10:**
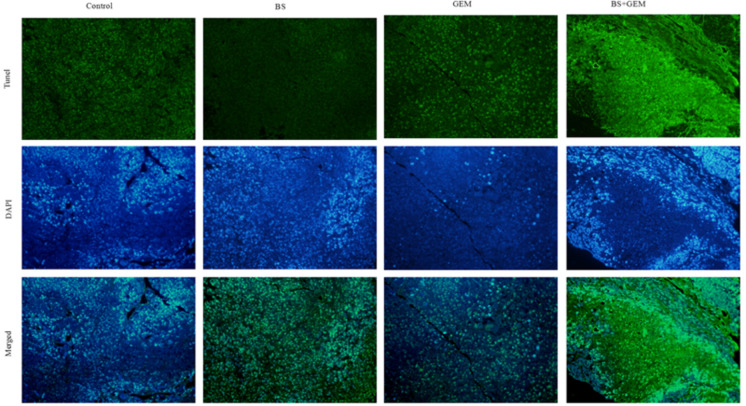
Mice tumor tissues by TUNEL assay.

To further explore the *in vivo* effects of BS and GEM treatments, tumor tissues were analyzed by Ki67, HE, and TUNEL staining and IHC. Tumor proliferation decreased significantly in the combination treatment group compared to either one of the agents treatment group, as indicated by lower Ki67 staining that demonstrated diminished cellular viability in the tumors (Figure 9A). Furthermore, necrosis of tumor cells increased significantly in the combination group compared to either one of the agents group, as indicated by HE staining (Figure 9A). The number of apoptotic cells also increased in the combination treatment group, as measured by TUNEL assay (Figure 10). Furthermore, we examined the levels of the protein NF-kB p-65, protein p-NF-kB p-65, pro-apoptotic protein Bax and anti-apoptotic protein Bcl-2 by IHC and western Blot. The IHC data showed that the combination treatment group significantly upregulated Bax levels but downregulated of Bcl-2 and p-NF-kB p-65 levels (Figures 9A,B), Moreover, the western blot showed that the combination treatment significantly upregulated Bax levels but downregulated Bcl-2 and p-NF-kB p-65 levels, whereas it exhibited no effect on the total level of NF-kB p-65 (Figures 9C–E). These results demonstrate that the combination of BS and GEM could significantly induce apoptosis in PC cells.

**FIGURE 11:**
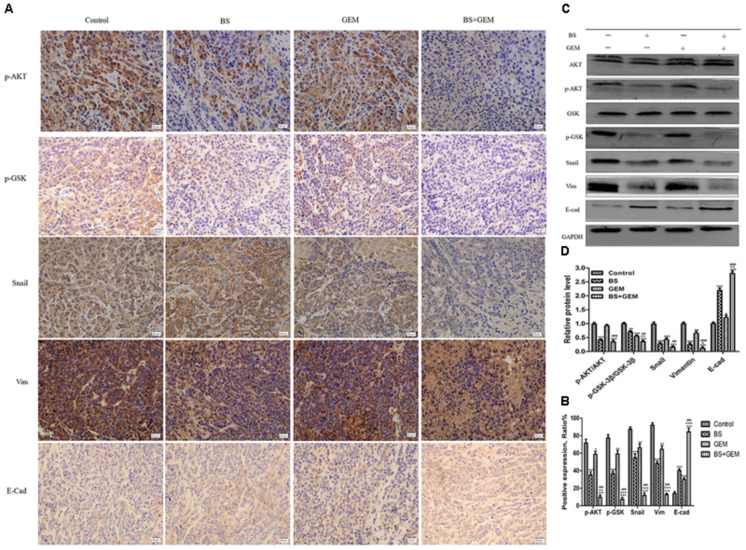
Combination of β-sitosterol (BS) and gemcitabine (GEM) downregulated the expression of epithelial–mesenchymal transition (EMT) markers and AKT/GSK3β signaling pathways in xenograft tissues. **(A,B)** Tumor tissues were immunohistochemically stained for determining p-Akt, p-GSK-3β, Snail, vimentin and *E*-cadherin levels, The relative levels of p-GSK-3β, p-AKT, Snail, vimentin, and *E*-cadherin were shown in the histograms. All data are depicted as mean ± SD (*n* = 3; ^∗^*P* < 0.05; ^∗∗^*P* < 0.01; ^∗∗∗^*P* < 0.001; ^+^*P* < 0.01; ^++^*P* < 0.001; ^+++^*P* < 0.001; ^###^*P* < 0.001. **(C,D)** Mice tumors were assessed by western blotting for determining Akt, p-Akt, GSK-3β, p-GSK-3β, Snail, vimentin, and *E*-cadherin expression, the relative protein levels of p-Akt / Akt, p-GSK-3β / GSK-3β, Snail, vimentin, and *E*-cadherin were shown in the histograms. All data are depicted as mean ± SD (*n* = 3; ^∗^*P* < 0.05; ^∗∗^*P* < 0.01; ^∗∗∗^*P* < 0.001; ^+^*P* < 0.01; ^++^*P* < 0.001; ^##^*P* < 0.01; ^###^*P* < 0.001).

**FIGURE 12:**
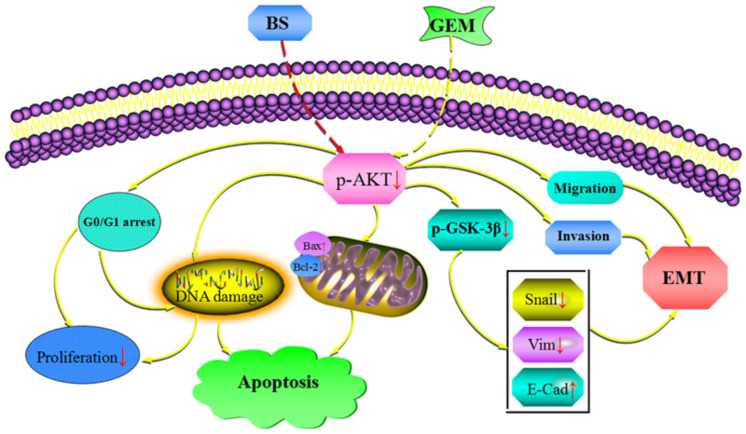
Combinationof β-sitosterol (BS) and gemcitabine (GEM) induced apoptosis and GO/G1 arrest and downregulated the expression of epithelial–mesenchymal transition (EMT) markers and AKT/GSK3β signaling pathways in pancreatic cancer cells.

We additionally investigated whether BS and GEM alone or in combination could downregulate the levels of EMT markers and Akt/GSK-3β pathway in the tumor tissue. IHC and western blotting revealed that the combination group exhibited increased expression of *E*-cadherin, whereas it significantly downregulated Snail and vimentin expression (Figures 11A–D). In addition, phospho-Akt and phospho-GSK-3β also decreased in the combination treatment group, whereas it exhibited no effect on the total level of AKT and GSK-3β in any of the treatment groups, which was consistent with the *in vitro* results (Figures 11A–D). Taken together, our *in vivo* results are consistent with our *in vitro* findings and collectively provide convincing evidence in support of the superior antitumor efficacy of the combination treatment with BS and GEM and may be indicated as a potential novel strategy for PC treatment (Figure 12).

## Discussion

PC remains one of the most lethal malignancies, despite the immense progress in chemotherapy and radiotherapy and is still highly resistant to all treatment options, including GEM. Therefore, there is an urgent demand to find novel reagents or combination therapy methods for treating PC to overcome the resistance to GEM. Here, we demonstrated that BS effectively inhibited cell viability and induced apoptosis and G0/G1 phase cell cycle arrest in PC cells. Moreover, BS downregulated the expression of EMT markers and the AKT/GSK3β signaling pathway in PC cells. More importantly, the combination of BS and GEM exhibited a significant synergistic effect compared with BS or GEM treatment alone both *in vivo* and *in vitro*.

This is the first report to show that BS alone and in combination with GEM exhibited a significantly inhibitory effect in PC cells and xenograft tumor. Drugs derived from plant sources have been widely and notably applied in cancer research in the past 20 years (Katiyar et al., 2012; Yoshida et al., 2017). A high number of phytochemicals have been confirmed to exhibit antitumor activities by inducing apoptosis in cancer cells (Millimouno et al., 2014). Apoptosis promotion in cancer cells is regarded as a promising chemotherapy strategy to treat cancer. In addition to their pro-apoptotic effect, molecular mechanism studies have also further elucidated that these phytochemicals target many important therapeutic signaling pathways in cancer cells (Gali-Muhtasib et al., 2015). Oncogenic kas mutation was found in more than 80% PC patients (Kang et al., 2018; Naiara et al., 2018). MIA-PaCa-2 was found kras mutation in G12C. In the contrast, BXPC-3 was kras wild type cell line. We chose the two cell lines to explore the anti-tumor effect of BS alone or combined with GEM in PC. BS, an important natural product secreted from Hedyotis diffusa Willd, suppressed cell proliferation in MIA-PaCa-2 and BXPC-3 cells in a dose- and time-dependent manner. Hoechst 33258 staining and flow cytometry also revealed an increased apoptosis rate in response to BS treatment. Furthermore, we examined the levels of the pro-apoptotic protein Bax, anti-apoptotic protein Bcl-2, protein NF-kB p-65 and protein p-NF-kB p-65 by western blot. These data suggested that BS significantly inhibited cell growth and promoted apoptosis of PC cells. Moreover, our results also showed that BS induced G0/G1 phase arrest in MIA-PaCa-2 and BXPC-3 cells, which may contribute to growth inhibition.

Drug combinations are important and widely applied to treat the most fatal of diseases, such as AIDS and cancer. The main objective for developing drug combinations is to design a synergistic therapeutic effect for reducing or delaying the induction of drug resistance and to achieve toxicity and dose reduction (Chou, 2010; Omar et al., 2016). Therefore, further assessment of the synergistic/additive/antagonistic effect of the combination treatment with GEM and BS was performed, and the CI values are shown in Figures 4C–F. From the results, the CI values were all < 1.00, indicating that the combination of GEM and BS synergistically inhibited proliferation of MIA-PaCa-2 and BXPC-3 cells. In addition, when treated MIA-PaCa-2 and BXPC-3 cells with 250 μM/L BS and 50 μM/L GEM, the CI values were 0.665 and 0.316, respectively, which exhibited the strongest synergistic effect than either one of the agents treatment group.

Apoptosis evasion is one of the significant mechanisms of PC cell resistance against GEM (McCubrey et al., 2015). Our study showed that the combination group induced a significantly higher apoptosis rate than that induced by groups treated with either one of the agents. Hoechst 33258 staining and flow cytometry analysis demonstrated that the combination treatment induced higher levels of apoptosis than that by either of the agents alone. To study the synergistic effect mechanism, we examined two genes that are regarded as the major regulators of apoptosis, the anti-apoptotic gene Bcl-2 and the pro-apoptotic gene Bax. Bax is a member of the Bcl-2 protein family related to apoptosis. The ratio of pro- and anti-apoptotic molecules modulates apoptosis (Knudson and Korsmeyer, 1997; Fan et al., 2018). The results illustrated that the combination treatment significantly downregulated Bcl-2 levels but upregulated Bax levels (Figures 7D,E). Comprehensively, the ratio of Bax to Bcl-2 increased in the combination treatment group. Moreover, the results proved that the combination of GEM and BS significantly induced G0/G1 phase arrest in cancer cells compared groups treated with GEM or BS alone. Taken together, the combination treatment group induced apoptosis and G0/G1 phase arrest more evidently in PC cells compared with either one of the agents treatment group.

The Akt signaling pathway is one of the main survival pathways in tumor cells, and its expression rate is frequently high in many cases of carcinoma, multiple pathological processes are involved with the dysregulation of this pathway, including proliferation, cell cycle, migration, invasion, angiogenesis, metastasis, tumorigenesis, and drug resistance (Chan et al., 2014; Zhang et al., 2015). GSK-3β, a serine/threonine kinase (Doble and Woodgett, 2003), is abnormally enriched in human PC cells and another downstream effector of AKT and can be phosphorylated and deactivated by AKT, its aggregation in the nucleus is related to tumor differentiation and kinase activity (Ougolkov et al., 2006; Zhang et al., 2015). Previous studies have confirmed that Akt/GSK-3β modulates the metastasis of different type of tumors by regulating EMT, such as gastric, lung, hepatocellular, breast carcinoma, head and neck squamous cell carcinoma and colorectal, prostate, bladder cancer (Zhang et al., 2013; Liu et al., 2014; Zhou et al., 2015). Moreover, many chemotherapeutic drugs have been got resistance by activating AKT, this mainly caused the failure of chemotherapy (Strouch et al., 2009; Massihnia et al., 2017; Meng et al., 2018). Meanwhile, a number of studies have been reported that p-AKT could be activated by GEM, which caused further development of EMT and the activation of NF-kB (Lee et al., 2008; Zhuang et al., 2017; Wang et al., 2018). Our results demonstrated that BS effectively reduced phospho-NF-kB p65, phospho-Akt, phospho-GSK-3β levels and EMT markers, more importantly, it exhibits a synergistic effect with GEM in PC.

In the present study, we demonstrated that BS alone and combined with GEM evidently suppressed migration and invasion by both MIA-PaCa-2 and BXPC-3 cells. Additionally, western blotting showed that BS and GEM significantly attenuated EMT by downregulating the expression of Snail and vimentin and upregulating the expression of *E*-cadherin in PC cells. Most remarkably, our *in vivo* study showed that combination treatment with GEM and BS remarkably inhibited tumor growth. In addition, IHC results also confirmed that BS inhibited phospho-Akt and phospho-GSK-3β levels and downregulated the expression of Snail and vimentin but upregulated the expression of E-cadherin. Tumor proliferation decreased evidently in the combination treatment group compared with either one of the agents treatment group, as indicated by lower Ki67 staining that demonstrated diminished cellular viability in the tumors. HE staining showed that necrosis of tumor cells increased significantly in the combination treatment group compared with either one of the agents treatment group. As indicated by the TUNEL assay, the combination treatment resulted in increased cell apoptosis compared to that in groups treated with either one of the agents. Furthermore, we examined the levels of the protein p-NF-kB p-65, pro-apoptotic protein Bax and anti-apoptotic protein Bcl-2 by IHC and western blot, and the data showed that the combination therapy significantly upregulated Bax levels but downregulated Bcl-2 and p-NF-kB p-65 levels. Taken together, these data suggested that the BS could effectively promote the chemosensitivity of GEM in PC. In addition, body weight and organ indexes indicated that BS is relatively safe for xenograft nude mice, indicating that BS is a safe and potential therapeutic anti-PC agent. Therefore, our research may provide an alternative strategy to treat PC.

## Conclusion

In conclusion, our study demonstrated the efficacy, for the first time, that β-sitosterol alone and combined with gemcitabine against human pancreatic cancer *in vivo* and *vitro*. Furthermore, the efficacy of GEM increased evidently when combined with BS. Therefore, our research may provide an alternative strategy to treat PC. However, drug sensitizers derived from natural plants warrant further research to assess their advantages and feasibility in clinical applications.

## Author Contributions

Z-qC and X-xW designed the experiments and wrote the manuscript. Z-qC, LL, J-wX, X-bL, Z-jM, A-cS, YW, and Y-jS accomplished the experiments and analyzed the data. X-xW, LL, and G-rZ supervised all the experiments and analyzed the data.

## Conflict of Interest Statement

The authors declare that the research was conducted in the absence of any commercial or financial relationships that could be construed as a potential conflict of interest.
