# Corrigendum: β-Sitosterol and Gemcitabine Exhibit Synergistic Anti-Pancreatic Cancer Activity by Modulating Apoptosis and Inhibiting Epithelial–Mesenchymal Transition by Deactivating Akt/GSK-3β Signaling

**DOI:** 10.3389/fphar.2020.565535

**Published:** 2020-11-20

**Authors:** Zhang-qi Cao, Xue-xi Wang, Li Lu, Jing-wen Xu, Xiao-bin Li, Guang-ru Zhang, Zhan-jun Ma, An-chen Shi, Yan Wang, Yu-jun Song

**Affiliations:** ^1^ School of Basic Medical Sciences, Lanzhou University, Lanzhou, China; ^2^ Qinghai Hospital of Traditional Chinese Medicine, Xining, China; ^3^ The Second Clinical School, Lanzhou University, Lanzhou, China

**Keywords:** β-sitosterol, gemcitabine, pancreatic cancer, apoptosis, EMT, AKT, GSK-3β

In the original article, there was a mistake in **Figures 2,3,6 and 7** as published. The incorrect images were erroneously inserted.

Firstly, the label of S and G2/M were marked reversed in Figures 2A,B and 6. Besides, one picture was mistakenly showed in Figure 6. In addition, due to the carelessness of the picture combination and image processing, in Figure 3A and Figure 7A,D, some pictures were mistakenly placed. The corrected Figures 2,3,6 and 7 appears below.

**FIGURE 2:**
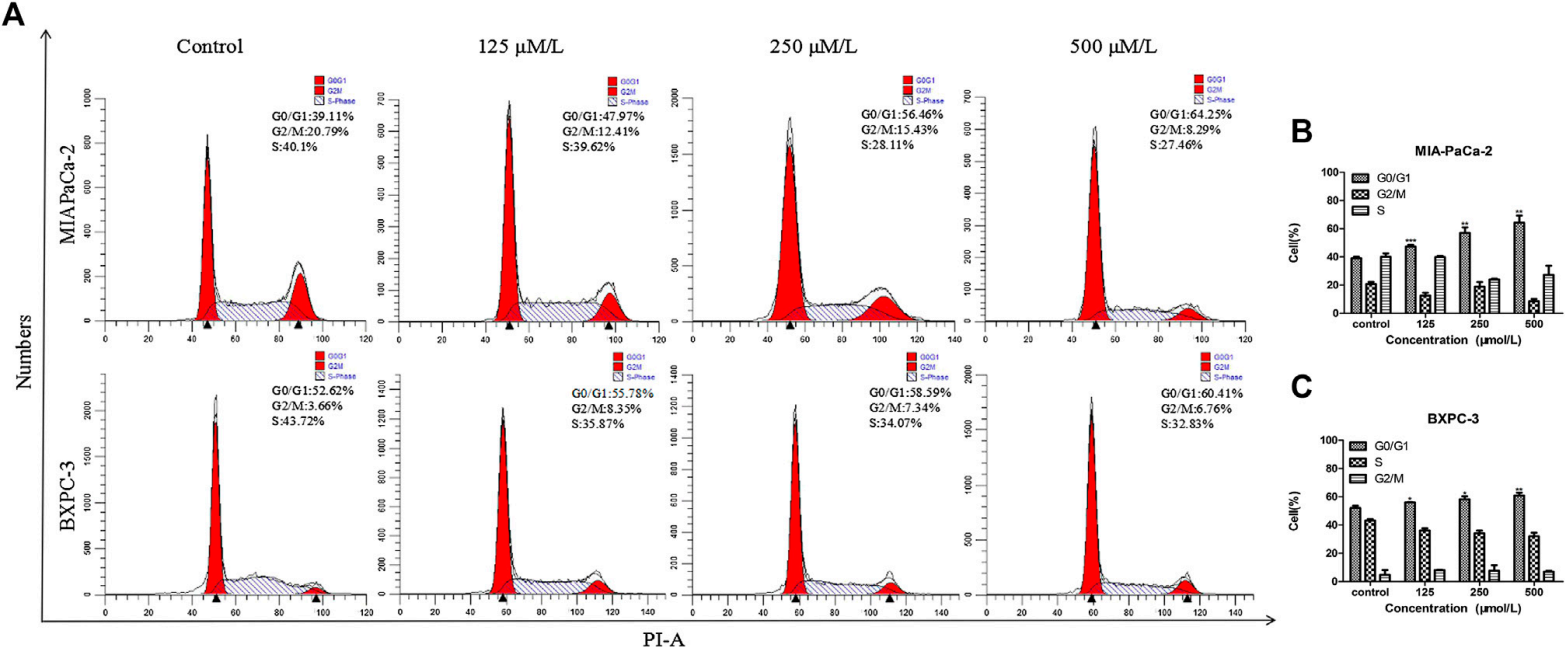
β-Sitosterol (BS) affects cell cycle progression in pancreatic cancer cells. **(A–C)** MIA-PaCa-2 and BXPC-3 cells were treated with different concentrations of BS for 48 h. G0/G1 cell cycle arrest were observed in MIA-PaCa-2 and BXPC-3 cells. All data are depicted as mean ± SD (*n* = 3; *p < 0.05; **p < 0.01; ***p < 0.001).

**FIGURE 3:**
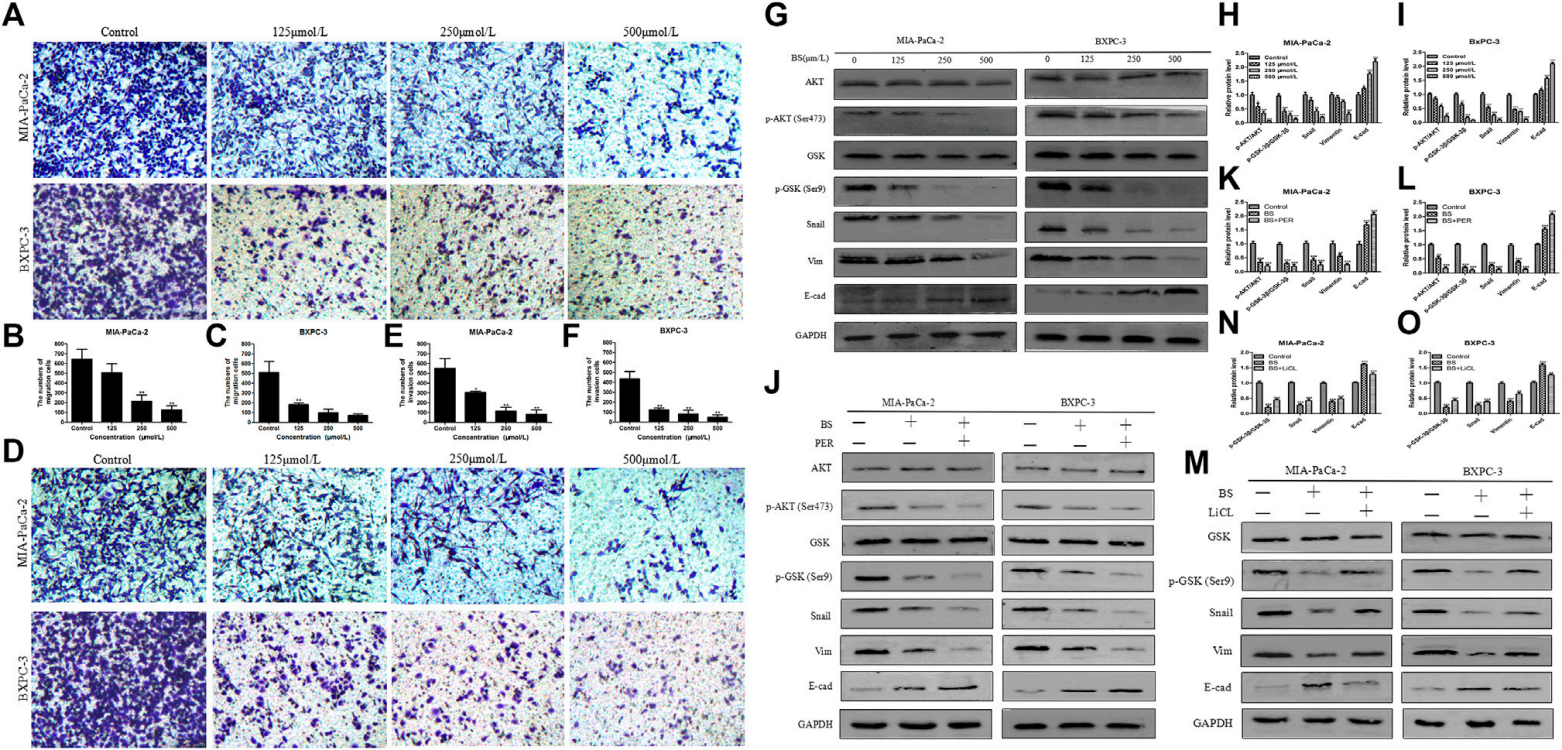
β-Sitosterol (BS) decreases migration and invasion and downregulates the expression of epithelial–mesenchymal transition (EMT) markers and AKT/GSK-3β signaling pathways in pancreatic cancer cells. **(A–C)** For transwell migration assays, MIA-PaCa-2 and BXPC-3 cells were treated with various concentrations of BS for 48 h. The number of cells were counted under a microscope (200× magnification). Quantification results are showed for migration of MIA-PaCa-2 and BXPC-3 cells. All data are depicted as mean ± SD (*n* = 3; *p < 0.05; **p < 0.01). **(D–F)** For Matrigel-coated invasion assays, MIA-PaCa-2 and BXPC-3 cells were treated with various concentrations of BS for 48 h. The number of cells were counted under a microscope (200× magnification). Quantification results are shown for invasion by MIA-PaCa-2 and BXPC-3 cells. All data are depicted as mean ± SD (*n* = 3; *p < 0.05; **p < 0.01). **(G–I)** MIA-PaCa-2 and BXPC-3 cells were treated with various concentrations of BS for 48 h, and the expression levels of Akt, p-Akt, GSK-3β, p-GSK-3β, Snail, vimentin, and E-cadherin were detected by western blotting. The relative protein levels of p-Akt/Akt, p-GSK-3β/GSK-3β, Snail, vimentin, and E-cadherin in MIA-PaCa-2 and BXPC-3 cells were shown in the histograms. All data are depicted as mean ± SD (*n* = 3; *p < 0.05; **p < 0.01; ***p < 0.001). **(J–L)** MIA-PaCa-2 and BXPC-3 cells were treated with just culture medium, BS (250 μM/L), or both BS (250 μM/L) and PER (10 μM/L). The expressions of Akt, p-Akt, GSK-3β, p-GSK-3β, Snail, vimentin, and E-cadherin in MIA-PaCa-2 and BXPC-3 cells were tested by western blotting, the relative protein levels of p-Akt/Akt, p-GSK-3β/GSK-3β, Snail, vimentin, and E-cadherin were shown in the histograms. All data are depicted as mean ± SD (*n* = 3; **p < 0.01; ***P < 0.001). **(M–O)** MIA-PaCa-2 and BXPC-3 cells were treated with just culture medium, BS (250 μM/L), or both BS (250 μM/L) and LiCL (20 mM/L). The expressions of GSK-3β, p-GSK-3β, Snail, vimentin, and E-cadherin in MIA-PaCa-2 and BXPC-3 cells were tested by western blotting, the relative protein levels of p-GSK-3β/GSK-3β, Snail, vimentin, and E-cadherin were shown in the histograms. All data are depicted as mean ± SD (*n* = 3; **p < 0.01; ***p < 0.001).

**FIGURE 6:**
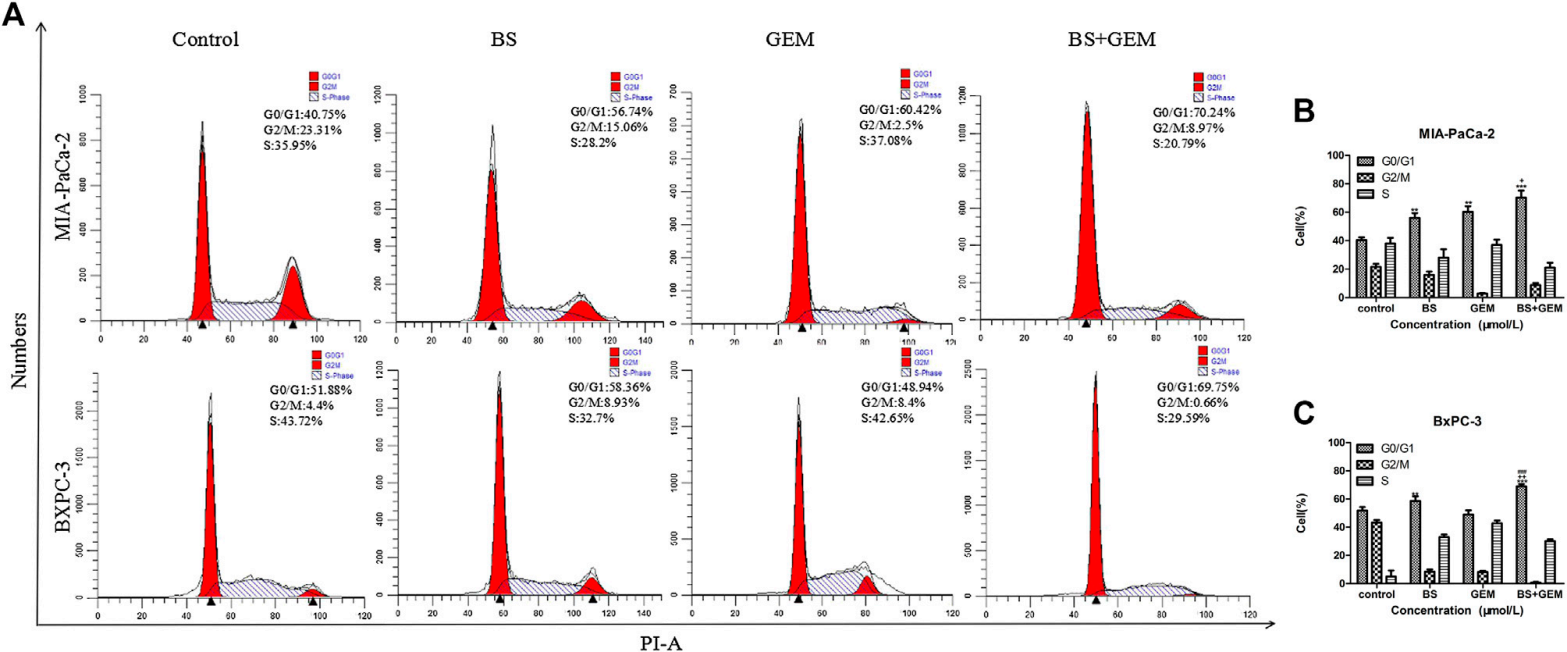
Combination of β-sitosterol (BS) and gemcitabine (GEM) affect cell cycle progression of pancreatic cancer cells. MIA-PaCa-2 and BXPC-3 cells were treated with BS (250 μM/L) and GEM (50 μM/L) alone and in combination for 48 h and analyzed by flow cytometry. **(A–C)** Cell cycle distribution in the G0/G1 phase was observed to be augmented in the combination group compared with either one of the agents group. All data are depicted as mean ± SD (*n* = 3; **p < 0.01; ***p < 0.001; ^+^ *p* < 0.05; ^++^ *p* < 0.001; ^###^ *p* < 0.001).

**FIGURE 7:**
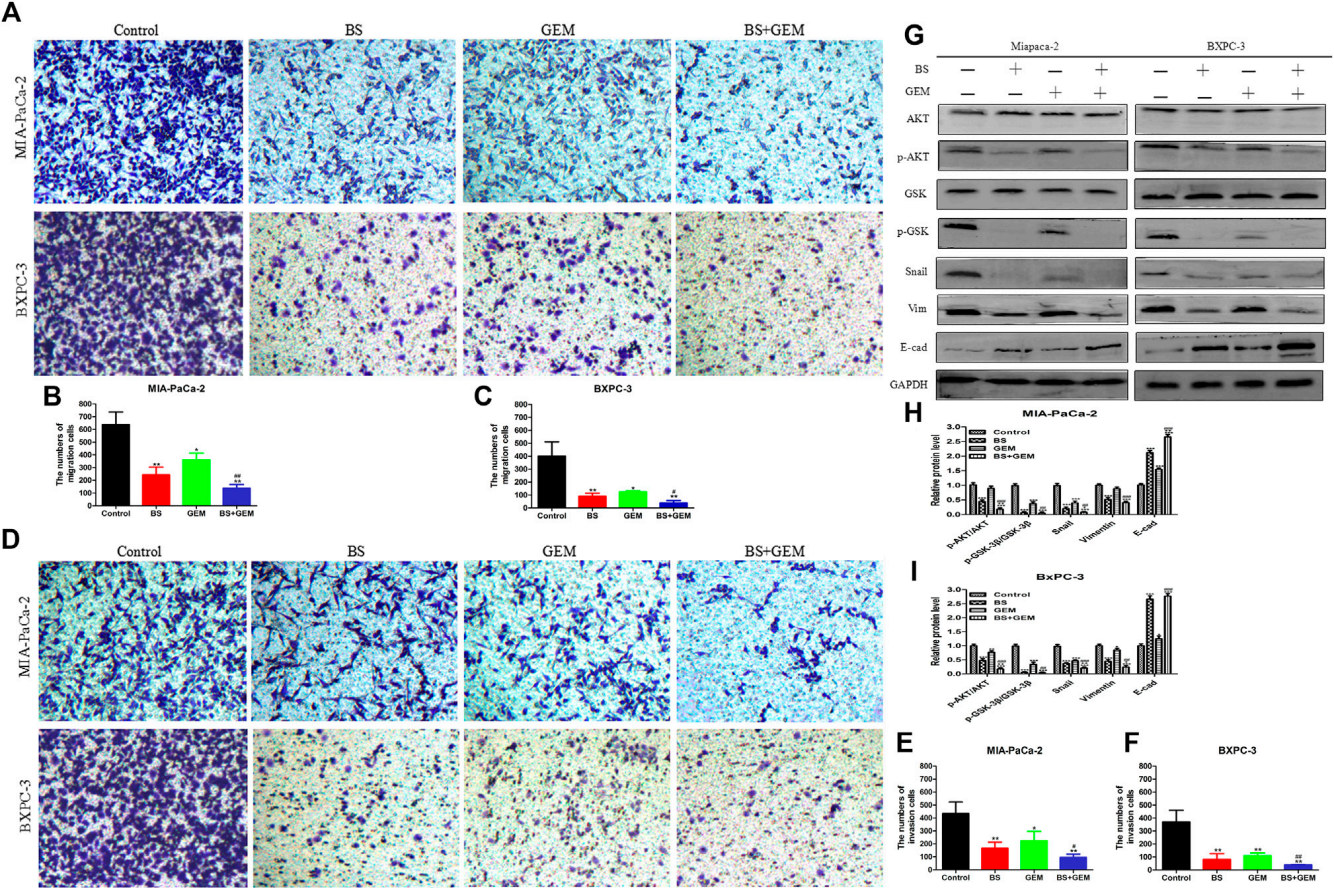
Combination of β-sitosterol (BS) and gemcitabine (GEM) synergistically decrease migration and invasion and downregulate the expression of epithelial–mesenchymal transition (EMT) markers and AKT/GSK-3β signaling pathways in pancreatic cancer cells. **(A–C)** For transwell migration assays, MIA-PaCa-2 and BXPC-3 cells were treated with BS (250 μM/L) and GEM (50 μM/L) alone and in combination for 48 h. The number of cells were counted under a microscope (200× magnification). Quantification results are shown for migration of MIA-PaCa-2 and BXPC-3 cells. All data are depicted as mean ± SD (*n* = 3; *p < 0.05; **p < 0.01; ^#^ *p* < 0.05; ^##^ *p* < 0.01). **(D–F)** For Matrigel-coated invasion assays, MIA-PaCa-2 and BXPC-3 cells were treated with BS (250 μM/L) and GEM (50 μM/L) alone and in combination for 48 h. The number of cells was counted under a microscope (200× magnification). Quantification results are shown for invasion by MIA-PaCa-2 and BXPC-3 cells. All data are depicted as mean ± SD (*n* = 3; *p < 0.05; **p < 0.01; ^#^ *p* < 0.05; ^##^ *p* < 0.01). **(G–I)** MIA-PaCa-2 and BXPC-3 cells were incubated with BS (250 μM/L) and GEM (50 μM/L) alone and in combination for 48 h. The expression levels of Akt, p-Akt, GSK-3β, p-GSK-3β, Snail, vimentin, and E-cadherin were detected by western blotting. the relative protein levels of p-Akt/Akt, p-GSK-3β/GSK-3β, Snail, vimentin, and E-cadherin were shown in the histograms. All data are depicted as mean ± SD (*n* = 3; *p < 0.05; **p < 0.01; ***p < 0.001; ^+^ *p* < 0.05; ^++^ *p* < 0.001; ^##^ *p* < 0.01; ^###^ *p* < 0.001).

The authors apologize for this error and state that this does not change the scientific conclusions of the article in any way. The original article has been updated.

